# Signatures of human regulatory T cells: an encounter with old friends and new players

**DOI:** 10.1186/gb-2006-7-7-r54

**Published:** 2006-07-12

**Authors:** Susanne Pfoertner, Andreas Jeron, Michael Probst-Kepper, Carlos A Guzman, Wiebke Hansen, Astrid M Westendorf, Tanja Toepfer, Andres J Schrader, Anke Franzke, Jan Buer, Robert Geffers

**Affiliations:** 1 Department of Mucosal Immunity, German Research Centre for Biotechnology, Braunschweig, Germany; 2 Volkswagen Foundation Junior Research Group, Department of Visceral and Transplant Surgery, Hanover Medical School, Hanover, Germany; 3 Department of Vaccinology, German Research Centre for Biotechnology, Braunschweig, Germany; 4 Department of Urology, Philipps-University Medical School, Marburg, Germany; 5 Department of Hematology and Oncology, Hanover Medical School, Hanover, Germany; 6 Institute of Medical Microbiology, Hanover Medical School, Hanover, Germany

## Abstract

**Background:**

Naturally occurring CD4^+^CD25^+ ^regulatory T cells (T_Reg_) are involved in the control of autoimmune diseases, transplantation tolerance, and anti-tumor immunity. Thus far, genomic studies on T_Reg _cells were restricted to murine systems, and requirements for their development, maintenance, and mode of action in humans are poorly defined.

**Results:**

To improve characterization of human T_Reg _cells, we compiled a unique microarray consisting of 350 T_Reg _cell associated genes (Human T_Reg _Chip) based on whole genome transcription data from human and mouse T_Reg _cells. T_Reg _cell specific gene signatures were created from 11 individual healthy donors. Statistical analysis identified 62 genes differentially expressed in T_Reg _cells, emphasizing some cross-species differences between mice and humans. Among them, several 'old friends' (including *FOXP3*, *CTLA4*, and *CCR7*) that are known to be involved in T_Reg _cell function were recovered. Strikingly, the vast majority of genes identified had not previously been associated with human T_Reg _cells (including *LGALS3*, *TIAF1*, and *TRAF1*). Most of these 'new players' however, have been described in the pathogenesis of autoimmunity. Real-time RT-PCR of selected genes validated our microarray results. Pathway analysis was applied to extract signaling modules underlying human T_Reg _cell function.

**Conclusion:**

The comprehensive set of genes reported here provides a defined starting point to unravel the unique characteristics of human T_Reg _cells. The Human T_Reg _Chip constructed and validated here is available to the scientific community and is a useful tool with which to study the molecular mechanisms that orchestrate T_Reg _cells under physiologic and diseased conditions.

## Abstract 2

Comparison of the gene expression in human T regulatory cells and naïve cells using a T regulatory cell-specific microarray reveals cell-specific gene signatures.

## Background

One of the most striking capacities of the immune system is its ability to discriminate between self and non-self, thereby avoiding autoimmune responses while allowing effective immunity against infections. Several mechanisms to maintain tolerance and immune homeostasis have evolved. On the one hand, self-reactive T cells are deleted during their development in the thymus in a process known as central tolerance. However, because this negative selection is incomplete, self-reactive T cells that have escaped from this clonal deletion must be controlled in the periphery. T_Reg _cells actively suppress activation and expansion of self-reactive escapees as part of a process termed peripheral tolerance [1]. Thus, T_Reg _cells control the delicate balance between immunity and tolerance, explaining their important role in autoimmune diseases, cancer, transplantation tolerance, and even allergy.

Several types of T_Reg _cells exist. Naturally occurring T_Reg _cells express the cell surface molecule CD25 (IL2RA) [2] and the transcriptional repressor FOXP3 (forkhead box P3), which is central for their development and function. These cells mature and migrate directly from the thymus and constitute approximately 2-3% of total human CD4^+ ^T cells [3-5]. Apart from these naturally occurring thymus-derived T_Reg _cells, antigen presentation by immature dendritic cells, IL-10, transforming growth factor-β, and possibly intrerferon-α possess the capability to convert naïve CD4^+^CD25^- ^or CD8^+^CD25^- ^T cells into regulatory T cells in the periphery [6-9]. These CD4^+ ^derived adaptive regulatory T cells are subdivided into T regulatory 1 (T_R_1) and T helper 3 (T_h_3) cells, according to their distinct cytokine profiles [10,11].

However, isolation of regulatory T cells remains difficult because the availability of specific marker molecules is still limited. Apart from CD25, additional surface molecules have been reported to be associated with T_Reg _cell function, such as cytotoxic T lymphocyte associated antigen (CTLA)4 [12], tumor necrosis factor receptor superfamily (TNFRSF) member 18 (or GITR) [13], and selectin L (SELL or CD62L) [14]. However, all of these molecules are also expressed by naïve CD4^+^CD25^- ^T cells upon activation, thereby hampering discrimination between regulatory and conventionally activated CD4^+ ^T cells. Furthermore, CD25 as well as other T_Reg _cell molecules (for instance, GITR and CTLA4) are not expressed on all CD4^+ ^T cells with regulatory function [15]. Recently, new genes such as neuropillin 1 (Nrp1) for mouse and CD27 coexpression with CD25 for human were suggested as useful markers to distinguish regulatory from effector T cells [16,17]. Like murine cells, human CD4^+^CD25^+ ^T_Reg _cells express significantly more FOXP3 mRNA and protein than do CD4^+^CD25^- ^T cells. However, in contrast to data obtained from mouse models, overexpression of FOXP3 in human CD4^+^CD25^- ^T cells alone is insufficient to generate potent suppressor T cells *in vitro*, suggesting that additional factors are required for the development, differentiation, and function of human T_Reg _cells [18].

Microarrays have illustrated their potential to unravel gene expression of various subsets of leukocytes. We and others have successfully used this technology to create signatures of murine regulatory T cells in different mouse models, contributing to a better understanding of the mechanisms underlying T_Reg _cell mediated tolerance and autoimmunity [16,19,20]. Thus far these genomic studies on T_Reg _cells have been restricted to murine systems. However, differences between humans and mice are highly suggestive and may present obstacles in the transfer from mouse models to actual human disease [21]. In this report we extend this approach to the characterization of human T_Reg _cells by studying 350 T_Reg _cell associated genes selected on the basis of whole-genome transcription data from human and mouse T_Reg _cells. Application of our nonredundant Human T_Reg _Chip to the study of highly purified CD4^+^CD25^+ ^T_Reg _cells and their naïve CD4^+^CD25^- ^counterparts isolated from peripheral blood of individual healthy donors revealed the presence of T_Reg _cell specific gene signatures. Combined with extensive pathway analysis, we provide a comprehensive set of genes to unravel the unique characteristics of human T_Reg _cells under physiological and diseased conditions.

## Results and discussion

### Development and validation of the Human T_Reg _Chip

Whole-genome expression data from human and mouse CD4^+^CD25^+ ^and CD4^+^CD25^- ^T cells, obtained using Affymetrix GeneChips (Affymetrix, Santa Clara, CA, USA), at the genomic scale were used to compile a primary list of genes involved in T_Reg _cell function. CD4^+ ^T cell subsets were isolated from either human peripheral blood or murine splenocytes and separated using FACS (fluorescence-activated cell sorting)-based cell sorting at purities consistently greater than 98%. Differential gene expression was determined using statistical parameters, as described under Material and methods, below. (For more detailed information, See Additional data file 1).

This primary data set from human T_Reg _cells was extended for genes that were affected by FOXP3 overexpression in cultured human CD4^+ ^T_h_cell lines. To this end, different CD4^+^CD25^- ^derived T_h _cell lines were generated by infection with retroviruses encoding for FOXP3 and GFP (green fluorescence protein) under the control of an internal ribosomal entry side (IRES) or with an empty control vector that contained only GFP. In these cells only FOXP3 overexpression could partially induce a T_Reg _phenotype *in vitro *(data not shown). Using Affymetrix GeneChips, these genetically engineered cells were compared with cells infected with T_h _GFP control vector. In addition, we also analyzed a human T_Reg _cell line derived from human CD4^+^CD25^+ ^T cells that maintained a regulatory phenotype *in vitro *and compared its gene expression profile with the control CD4^+ ^T_h _cell line. For the development of the Human T_Reg _Chip we included those genes in our primary data set that were differentially expressed in both experiments by more than twofold. (For more detailed information, see Additional data file 2).

In additionally, T_Reg _cell associated genes identified by literature search were also included (Additional data file 3). In summary, this resulted in the selection of 350 genes that were arranged on an oligonucleotide microarray. Furthermore, 45 control genes were included in the primary microarray design.

**Figure 1:**
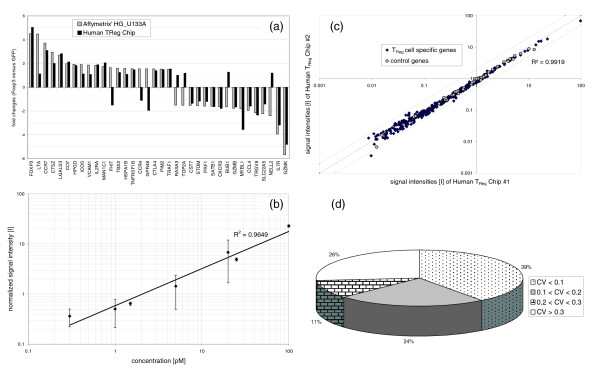
Performance of the Human T_Reg _Chip. (**a**) Comparability to Affymetrix. Splitted samples (FOXP3 or GFP transfected T cells) were hybridized to Affymetrix HG_U133A microarrays and Human T_Reg _Chips, respectively. Differentially expressed genes on the Affymetrix platform (regulation of at least 1.5-fold based on significant signal) were compared with those significant fold changes arising from the Human T_Reg _Chip platform. As demonstrated, 29 out of 36 genes exhibited similar regulation on the Human T_Reg _Chip compared with Affymetrix, resulting in a correlation of 81%. (**b**) Hybridization controls. Normalized signal intensities versus concentration of used hybridization controls are plotted as means of 5 (1.5 pmol/l, 25 pmol/l and 100 pmol/l) and 59 experiments applying the Human T_Reg _Chip. Standard deviations are indicated by error bars. Linear regression yields a correlation coefficient of >0.96 demonstrating a linear hybridization process covering more than three orders of magnitude of concentrations. (**c**) Reproducibility of the Human T_Reg _Chip. The same sample was hybridized to several Human T_Reg _Chips. A log-log plot of normalized signal intensities of two example selected slides is illustrated, showing that 99.7% of all signals are located along the bisecting line within the twofold range, reflecting low measurement noise in the data, even for low signal intensities. (**d**) Coefficients of variation (CV). The ratios of standard deviation and mean were calculated for each gene probed in eight replicates per microarray. CVs of all 59 experiments applying the Human T_Reg _Chip contributing to the expression profile of human T_Reg _cells are presented as means. As demonstrated, 73% of all signals have a CV below 0.3.

To obtain accurate and reliable transcription profiles, we validated the Human T_Reg _Chip in terms of cross-platform comparability, sensitivity, and reproducibility of measurements. Relative expression data gained from the experiments investigating FOXP3 affected gene expression on Affymetrix GeneChips, as described above, were used as reference data in a cross-platform evaluation. Therefore, identical samples, obtained either from FOXP3 infected CD4^+^CD25^- ^T cells or GFP expressing controls, were also hybridized to the Human T_Reg _Chip. Concordance of significantly regulated genes generated with the Human T_Reg _Chip and the reference data was 81% (29/36; Figure 1a). Opposite regulation was observed only for a few marginally regulated genes (7/36). The Affymetrix GeneChip data for the 350 genes included in the Human T_Reg _Chip is given in Additional data file 4). Furthermore, bacterial control genes at different concentrations were used to monitor microarray system sensitivity and the spectrum of linear signal measurement. A final concentration of 0.3 pmol/l was detectable, corresponding to approximately one transcript in 500,000 or approximately one copy per cell. Furthermore, we could demonstrate a linear regression between signal intensity and concentration covering more than three orders of magnitude (Figure 1b). To assess reproducibility, identical samples were applied to different Human T_Reg _Chips and signal intensities were compared among each other (Figure 1c). The median correlation coefficient obtained from 52 log-log-plots was 0.98, which is well in line with commercially available microarray formats [22,23] Finally, we determined the accuracy of measurements expressed as coefficient of variance calculated across eight replicates per gene. As depicted in Figure 1d, the vast majority of signal intensities (73%) calculated for the entire data set varied by less than 30%, reflecting the robustness of the applied microarray approach.

### Gene regulation in CD4^+^CD25^+ ^T_Reg _cells

To obtain accurate and reliable individual transcription profiles we isolated CD4^+^CD25^+ ^regulatory and CD4^+^CD25^- ^naïve T cells from peripheral blood of 11 healthy donors using MACS (Magnetic Cell Sorting) technology (Table 1). To estimate the fraction of T_Reg _cells in the CD4^+^CD25^+ ^cell population, we performed intracellular FOXP3 staining. Approximately 80% of the CD4^+^CD25^+ ^T cells were FOXP3 positive and exhibited regulatory T cell function *in vitro *(Additional data file 5). Each sample was measured in at least two independent microarray experiments. Using Statistical Analysis of Microarrays (SAM) analysis, we identified 62 genes significantly differentially expressed in regulatory compared to naïve T cells. Based on Gene Ontology and references in the literature, genes were classified into functional categories such as cytokines/chemokines and their receptors (12 genes), cell cycle and proliferation (11), apoptosis (7), signal transduction (9), and transcriptional regulation (10). A detailed description of these genes is summarized in Table 2. Among them, *LGALS3*, *CCR7*, *IL2RA *(*CD25*), *CTLA4*, *TRAF1*, *SATB1*, and *GZMK *were additionally found to be affected by retroviral overexpression of FOXP3 in CD4^+ ^T_h _cells (Figure 1a).

**Figure 2:**
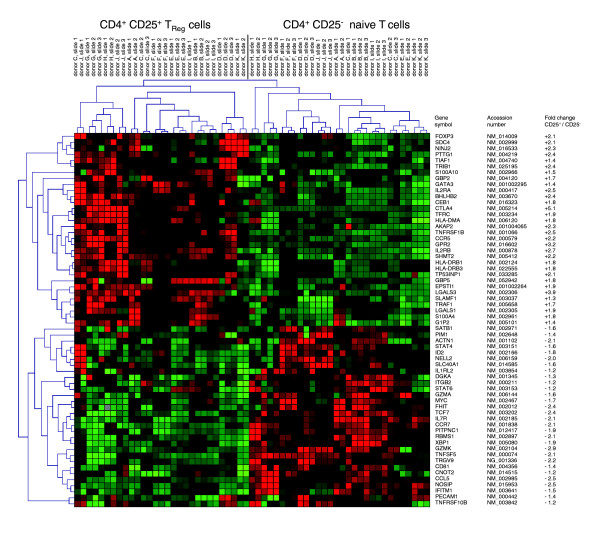
Transcriptional profiling of CD4^+^CD25^+ ^T_Reg _and CD4^+^CD25^- ^naïve T cells. To identify molecular differences between regulatory and naïve human T cells, differential expression of 350 genes was investigated by application of our Human T_Reg _Chip. Following data normalization, Statistical Analysis of Microarrays (SAM) was applied as a data mining tool to ascertain gene expression changes, identifying 62 significantly altered genes between both T cell subpopulations (delta = 2.46, median FDR [false discovery rate] = 0.48). After entering the generated data set into Genesis software, a two-dimensional hierarchical clustering analysis yielded the displayed transcriptional pattern, which discriminates between human regulatory and naïve T cells, and consists of 32 upregulated and 30 downregulated genes. Each row represents a gene probed on the Human T_Reg _Chip; each column shows expression of the 62 genes measured for each individual in the study. Red indicates genes that are expressed at higher levels compared with the mean signal intensities of all experiments, whereas downregulated genes are colored in green and black indicates signal intensities near the mean expression level.

Two-dimensional hierarchical clustering analysis was applied to arrange coexpressed genes and replicated experiments next to each other (Figure 2). The transcriptional pattern clearly separated CD4^+^CD25^+ ^regulatory from CD4^+^CD25^- ^naïve T cells and distinguished between 32 upregulated and 30 downregulated genes.

**Figure 3:**
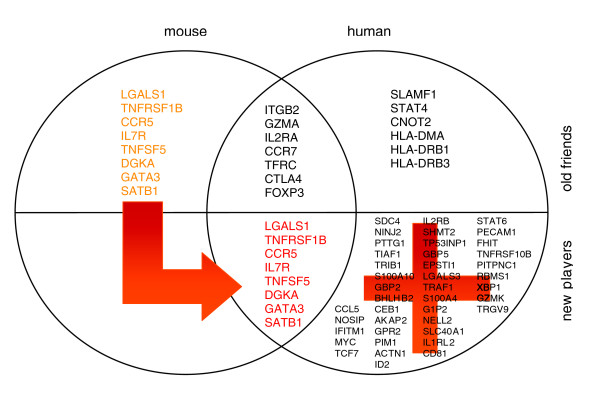
Old friends and new players. Genes differentially expressed in regulatory and naïve T cells, as identified by application of the Human T_Reg _Chip. The upper half of the Venn diagram summarizes 'old friends'(namely, T_Reg _cell associated genes that have previously been described in literature for either mouse or human). The lower half of the chart illustrates the new situation by showing all of the 'new players' of the T_Reg _cell fingerprint. As demonstrated by the extended intersection, we identified eight genes, which formerly had only been implicated in mouse T_Reg _cell immunology, as playing an additional role in human T_Reg_cell activity (red arrow). Furthermore, our results expanded our knowledge on the transcriptional pattern characterizing human T_Reg _cells by adding 41 new candidate genes (indicated by the red '+').

Twenty-one of these 62 genes have already been described in the literature as being associated with T_Reg _cells of both mouse and human origin, including *FOXP3*, *CTLA4*, *IL2RA *(*CD25*), and *ITGB2 *(Figure 3). Recovery of these 'old friends' confirmed our nonredundant microarray approach, including our cell separation strategy. Among the 62 genes, eight that were previously only implicated in murine T_Reg _cell biology were also detected as being differentially expressed in human T_Reg _cells (*LGALS1*, *IL7R*, *GATA3*, *SATB1*, *TNFRSF1B*, *TNSF5*, *DGKA*, and *CCR5*). Altogether, 15 genes were identified that were similarly regulated in mouse and human. Those genes at the intersection of both organisms reflect high levels of interspecies conservation during the evolutionary process, thereby lending credibility to their important role in T_Reg _cell development and function (Figure 3). In addition to *FOXP3*, *CTLA4 *and *IL2RA*, we also found the chemokine receptor 7 (*CCR7*), the transferring receptor (*TFRC*) and integrin beta 2 (*ITGB2*) genes in this intersection group between mouse and human. Furthermore, six genes previously associated with human T_Reg _cells were identified. Apart from the 'old friends', we identified 41 'new players' that have not previously been reported in the context of human T_Reg _cells (Figure 3).

**Figure 4:**
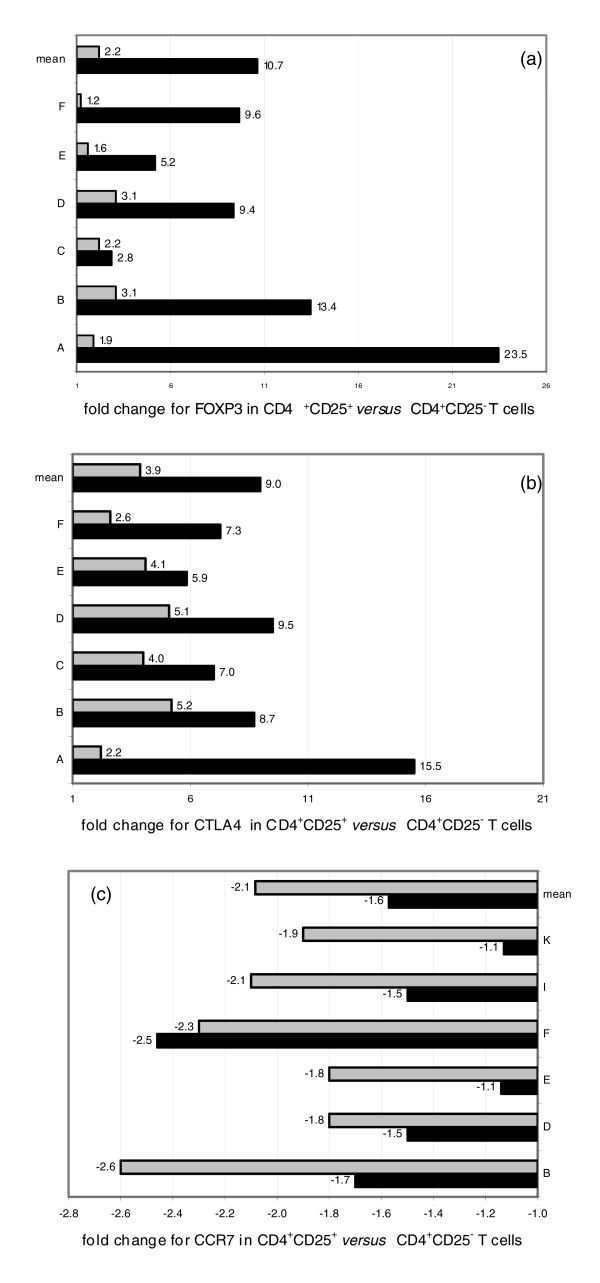
Old friends: confirmation of microarray results. Real-time RT-PCR was performed for (**a**) FOXP3, (**b**) CTLA4, (**c**) CCR7, and RPS9 (data not shown) expression in MACS separated human CD4^+^CD25^+ ^T_Reg _and CD4^+^CD25^- ^naïve T cells. Following normalization to RPS9, relative mRNA amounts in CD4^+^CD25^+ ^T_Reg _cells were adjusted to corresponding expression levels in CD4^+^CD25^- ^naïve T cells and expressed as fold changes. Real-time RT-PCR results, indicated by black bars, were compared with fold changes arising from the Human T_Reg _Chip (represented by grey bars). The healthy donors, randomly chosen, are specified by letters (see Table 1). RT-PCR, reverse transcription polymerase chain reaction.

**Figure 5:**
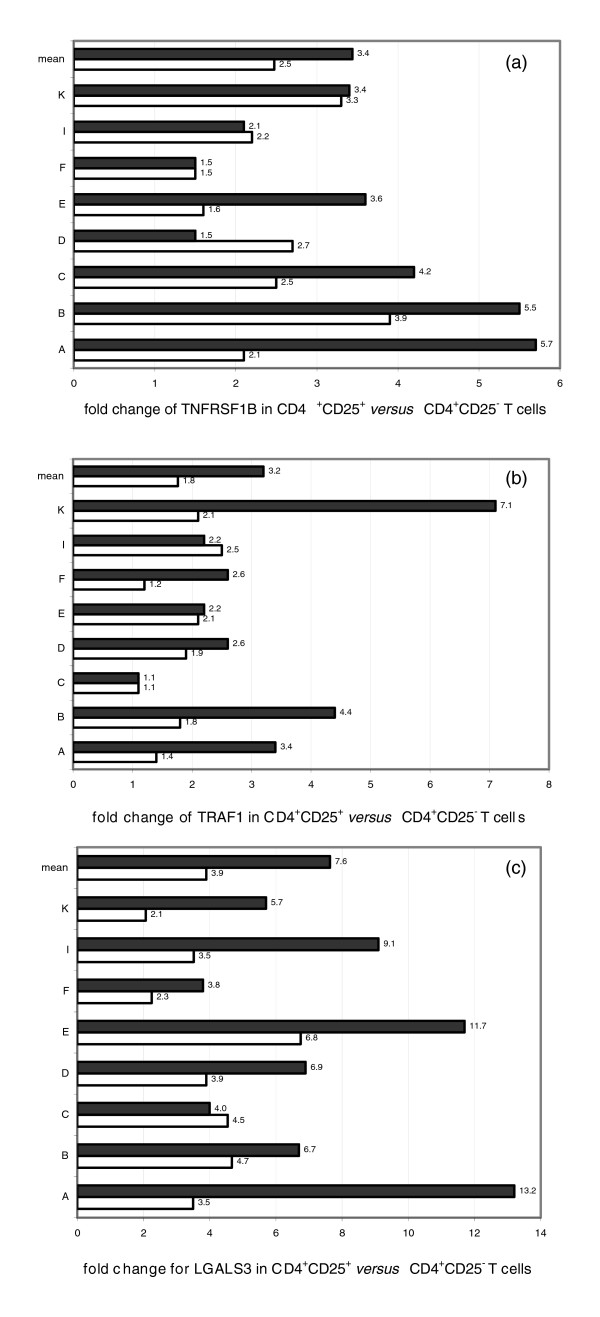
New players: confirmation of microarray results. Real-time RT-PCR was performed for (**a**) TNFRSF1B, (**b**) TRAF1, and (**c**) LGALS3 expression in MACS isolated human CD4^+^CD25^+ ^T_Reg _and CD4^+^CD25^- ^naïve T cells. Fold changes were calculated as described for Figure 4. Real-time RT-PCR results (black bars) were compared with fold changes arising from the Human T_Reg _Chip (white bars). The healthy donors are specified by letters (see Table 1). RT-PCR, reverse transcription polymerase chain reaction.

To verify the accuracy of our microarray data in more detail, real-time RT-PCR (reverse transcription polymerase chain reaction) was performed using the original samples. Referring to well characterized T_Reg _cell genes (*FOXP3*, *CTLA4*, and *CCR7*), we were able to confirm our approach (Figure 4). This gave greater credence and reliability to the numerous additional genes that have not yet been reported in T_Reg _cells. We selected three of these 'new players' (*TNFRSF1B*, *TRAF1*, *LGALS3*) and confirmed their T_Reg _cell specific expression by quantitative real-time RT-PCR (Figure 5). As shown, in general PCR results correlated well with the differential gene expression data obtained by application of the Human T_Reg _Chip. For a few donors variability in gene expression was observed between microarray and quantitative RT-PCR data, but the direction of change was consistent, lending confidence to the reliability of the Human T_Reg _Chip results. Quantitative differences in fold changes have previously been described; in particular, an underestimation of real expression changes by microarray approach versus quantitative RT-PCR has been reported [24,25].

### Signaling modules in T_Reg _cells

To elucidate potential pathway modules implicated in T_Reg _cell biology, we applied PathwayAssist, (Ariadne Genomics, Rockville, MD, USA), software to our unique expression dataset of human T_Reg _cells from individual healthy donors. Mapping the 62 T_Reg _cell specific genes yielded a network of 31 genes directly interacting with each other (data not shown). These 31 genes provided a comprehensive framework for further dissection into functional modules. These modules point to mechanisms controlling diverse cellular processes such as survival/apoptosis, T cell receptor signaling/activation/proliferation, and differentiation/maintenance of human T_Reg _cells and are described in the following text.

#### Genes controlling survival/apoptosis of T_Reg _cells

**Figure 6:**
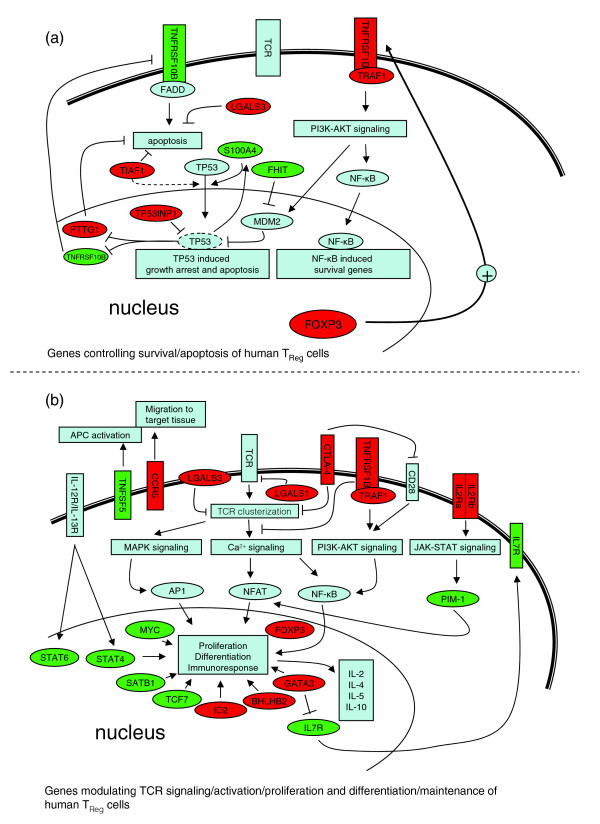
Functional dissection of signaling modules in human T_Reg _cells. Schematic representation of potential signaling pathways involving genes that control (**a**) survival/apoptosis, and (**b**) TCR signaling/activation/proliferation and differentiation/maintenance of human regulatory T cells, thereby mediating T_Reg _cell functionality. Transcriptional upregulation of genes in T_Reg _versus naïve T cell is marked by red symbols, whereas green symbols represent downregulated genes. Symbols filled with grey depict unaffected genes or summarize pathway modules.

Naturally occurring T_Reg _cells survive clonal deletion during their development in the thymus by escape from activation-induced cell death. This protective mechanism appears to be maintained in T_Reg _cells encountered in the periphery because we could identify a signaling module that counteracts apoptosis and mediates the release of survival factors (Figure 6a).

We found that FOXP3 induced upregulation of tumor necrosis factor receptor superfamily, member 1B (*TNFRSF1B*, *TNF*-*RII*) upon retroviral overexpression in CD4^+ ^T_h _cells (Figure 1a). *TNFRSF1B *was also upregulated in the *ex vivo *isolated CD4^+^CD25^+ ^T_Reg _cells from individual healthy donors (Figure 2). TNFRSF1B belongs to a group of transmembrane TNF receptor molecules characterized by TNF receptor-associated factor (TRAF)-interacting motifs (TIMs). Activation of TIM-containing TNF receptors leads to the recruitment of TRAF family members and subsequent activation of signal transduction pathways such as nuclear factor (NF)-κB, JNK, p38, ERK (extracellular signal-regulated kinase), and PI3K (phosphoinositide 3-kinase), which in turn influence immune responses and increase the expression of survival factors [26,27]. In accordance, we also found a significant upregulation of *TRAF1 *inboth FOXP3 transduced CD4^+ ^T_h _cells and *ex vivo *isolated human CD4^+^CD25^+ ^T_Reg _cells.

This mechanism is linked to additional molecules that control the nuclear translocation and, consequently, activity of TP53 (tumor protein p53), a tumor suppressor gene that induces cell growth arrest or apoptosis [28]. Although TIAF1 (TGFB-1 induced antiapoptotic factor 1) interacts with TP53 in the cytosol and may participate in its nuclear translocation, TP53INP1 (TP53 inducible nuclear protein 1) is engaged in the regulation of TP53 activity in the nucleus [29,30]. Both *TP53INP1 *and *TIAF1 *genes were found to be overexpressed in the naturally occurring T_Reg _cells in our study. Apart from this, *TIAF1 *is known to be upregulated in T_h_2 compared with T_h_1 lymphocytes, and a functional role as an apoptosis protector has been discussed [31].

We also identified *S100A4 *as being upregulated in the naturally occurring T_Reg _cells from our individual donors. S100A4 is a member of the S100 family of proteins containing two EF hand calcium binding motifs. EF-hands are helix-loop-helix motifs where the loop potentially binds Ca^2+^. Its expression is TP53 dependent and S100A4 is involved in the regulation of cell cycle progression and differentiation. Together with S100B, S100A4 is hypothesized to control tetramerization of TP53, leading to its nuclear translocation [32,33]. TP53 can activate the extrinsic apoptotic pathway through the induction of TNF receptor family members such as FAS and TNFRSF10B [28,34]. Both TNF receptors are characterized by their cytoplasmic death domain, which is responsible for transmission of apoptotic signals. Activation of these receptors leads to recruitment of intracellular death domain, containing adaptors such as FAS-associated death domain (FADD) and TNFR associated death domain (TRADD). These molecules activate the caspase cascade and subsequently induce apoptosis. The death domain clearly separates these TNF receptors from TNFRSF1B [26]. As a potential consequence of the assumed TP53 inactivation in T_Reg _cells, *TNFRSF10B *expression could be impaired.

Further evidence supporting this assumption was provided by another direct target of TP53. Expression of *PTTG1 *(pituitary tumor-transforming 1), which we found to be upregulated in our naturally occurring T_Reg _cells, can be directly repressed by activated TP53 in colorectal cancer cells. RNAi mediated knockdown of *PTTG1 *was sufficient to induce apoptosis, suggesting that repression of novel antiapoptotic genes by active TP53 can significantly contribute to apoptosis [34]. Controversially, it has been reported that PTTG1 can activate TP53 and BAX to increase apoptotic function, but this seems to be rather an indirect effect of PTTG1 and is dependent on other factors, such as *MYC*, which we found to be downregulated in the naturally occurring human T_Reg _cells [35]. Interestingly, c-*MYC *is a direct downstream target of PTTG1, which is part of the DNA-binding complex formed near the transcription initiation site of the c-*MYC *promoter [36].

We have detected additional genes that are downregulated in human T_Reg _cells, affecting the activation status of TP53. In lung cancer cells, it was shown that FHIT (fragile histidine triad gene) mediates MDM2 inactivation. The antiapoptotic molecule MDM2 is activated through the PI3K-AKT pathway, leading to inactivation of TP53 [37]. Thus, downregulation of *FHIT *also contributes to the inactive status of TP53.

Based on our data, we suggest that destabilization and thereby inactivation of TP53 provokes a shift in T_Reg _cells from apoptotic sensitivity to protection and survival. It is tempting to speculate that this mechanism allows T_Reg _cells to survive upon reactivation, whereas effector T cells underlie activation-induced cell death. This apoptotic process eliminates the expanded pool of effector lymphocytes during the contraction phase of the immune response and maintains lymphocyte homeostasis. In accordance with our findings, murine T_Reg _cells were reported to be more resistant to apoptosis when treated with dexamethasone or anti-CD95 antibody than CD4^+ ^total or CD4^+^CD25^- ^effector T cells [38,39]. Moreover, Fritzsching [40] and Wang [41] and their groups demonstrated that human T_Reg _cells are less sensitive to activation-induced cell death than their naïve counterparts. Galectin-3 (*LGALS3*) is one of the best characterized members of the evolutionary conserved family of galectins and was found to be strongly upregulated in our *ex vivo *isolated T_Reg _cells (Figure 2). In addition, *LGALS3 *was also induced upon FOXP3 overexpression in CD4^+ ^T_h _cells (Figure 1a). This is of special interest because LGALS3 is known to participate in apoptosis control. Whereas its secretion triggers apoptotic signal cascades in T cells [42], intracellular expressed LGALS3 acts as an antiapoptotic molecule [43-45]. The underlying mechanism was revealed in macrophages, suggesting that LGALS3 may prevent alterations of the mitochondrial membrane and formation of reactive oxygen species. Moreover, it has been reported that LGALS3 phosphorylation is necessary for its antiapoptotic activity. The increased expression level of LGALS3 further supports our idea of a shifted balance toward survival and fitness of T_Reg _cells.

#### Genes controlling T cell receptor signaling, activation, and proliferation of T_Reg _cells

The second module that was revealed in the present study involves genes controlling T cell receptor signaling, activation, and proliferation of human T_Reg _cells (Figure 6b). LGALS1 antagonizes T cell activation by partial phosphorylation of the T cell receptor (TCR)-ζ chain [46], can block secretion of proinflammatory cytokines such as IL-2, and skews the balance towards a T_h_2-type cytokine profile [47,48]. Dimeric LGALS1 triggers immunosuppressive IL-10 production in T cells, contributing to their immune regulatory function [49]. LGALS3 can potentially form complexes on the TCR with N-glycans, thereby limiting the lateral mobility of the TCR and resulting in restricted TCR-mediated signaling on T cells [42]. We therefore suggest that upregulation of both galectins in T_Reg _cells results in a modulation of their cytokine profile, thereby allowing appropriate regulation of effector cells and immune cell homeostasis.

This module also identified a set of genes, including *CTLA4*, *TNFRSF1B*, and *PIM1*, that controls proliferation (Figure 6b). CTLA4 plays a major role in inhibiting proliferation of T_Reg_cells. It is an activation-induced homo-dimeric glycoprotein receptor on T cells that interacts with the B7 ligands on the surface of antigen-presenting cells (APCs). The mechanism of T cell inactivation involves antagonism of CD28-dependent costimulation and direct negative signaling through its cytoplasmic tail. When engaged by B7, CTLA4 plays a key role as a negative regulator of T cell activation through down-regulation of cytokine production by preventing the accumulation of activator protein (AP)-1, NF-κB, and NFAT (nuclear factor of activated T-cells) in the nucleus. *CTLA4 *was found to be upregulated in our human T_Reg _cells. Its expression has been linked to enhanced suppressor activity and higher expression of FOXP3 in human T_Reg _cells. However, the blockade of CTLA4 resulted in a significant but incomplete loss of suppressor activity [50]. In addition to *CTLA4*, *TNFRSF1B *was also found to be upregulated in the human T_Reg _cells. TNFRSF1B is known to costimulate TCR-mediated activation in human T cells, thereby inducing activation markers, such as CD25. In contrast to CD28 costimulation, crosslinking of TNFRSF1B triggers different signaling pathways resulting in a modified cytokine profile. TNFRSF1B has the capacity to downregulate early TCR/CD28 induced calcium mobilization and inhibits T cell functions such as IL-2 and IL-10 production [51]. Compared with activated naïve T cells, the proliferation of T_Reg _cells in response to IL-2 is quite low, although the receptor for this cytokine is significantly upregulated. We could identify a serine/threoninekinase called PIM1 that directly transactivates NFAT at the end of the Ras signaling cascade to facilitate IL-2 dependent proliferation and/or survival of lymphoid cells. Furthermore, PIM1 enhances NFAT-dependent transactivation and IL-2 production in Jurkat T cells [52]. Because *PIM1 *is downregulated in T_Reg _cells from individual healthy donors, we propose a reduced signal transmission to NFAT mediating less responsiveness to IL-2 resulting in lower proliferation of T_Reg _cells.

#### Genes controlling differentiation and maintenance of T_Reg _cells

A third module extracted by our pathway analysis involves genes controlling T_Reg _cell differentiation and maintenance upon maturation in the thymus (Figure 6b). The differentiation of naïve T cells is induced by TCR activation and either IL-12/STAT (signal transducer and activator of transcription)4 or IL-4/STAT6 signaling pathways leading to a T_h_1/T_h_2 lineage specification that is further directed by the transcription factors T-bet and GATA3, respectively. *STAT4 *and *STAT6 *were both downregulated in the peripheral T_Reg _cells, indicating a potential inability to be transformed into T_h _cells upon restimulation via their TCR (Figure 2). Coexpression of *GATA3 *and *FOXP3*, but the lack of *T-bet*, suggests similarities in the gene expression profiles of T_h_2 and T_Reg _cells in humans.

In a recent study, transcription profiles of T_h_1 and T_h_2 cells isolated from human cord blood were analyzed. Although the overall concordance to our T_Reg _cell data set is quite low, we were able to detect a few genes similarly regulated in T_h_2 and T_Reg _versus naïve T cells (*TCF7*, *GZMA*, S100 family members). However, a few genes exhibited opposite expression behavior in T_h_2 cells compared with the T_Reg _cells (*SATB1 *and *ACTN1 *were upregulated in T_h_2 and down-regulated in T_Reg _cells). SATB1 and TCF7 are transcription factors that are functionally similar to GATA3 and have important functions in early thymocyte development [53,54]. For genes that were differentially expressed in T_h_1 versus naïve T cells, we found no similarities to our T_Reg _cell data set [55]. In summary, these data underline the concept that, like their murine homologs, human T_Reg _cells represent a separate lineage. They are undergoing a unique differentiation pathway distinct from those committing T_h_1 or T_h_2 cells, and are therefore equipped with a tightly regulated set of transcription factors acting in addition to FOXP3.

Another important question is how T_Reg _cell populations are regulated and maintained in the periphery. There is growing evidence favoring IL-7 as a master regulator of T cell homeostasis, based on its essential role in the homeostatic expansion of naïve T cells in response to low affinity antigens and its capacity to enhance expansion of peripheral T cells dramatically in response to high affinity antigens [56]. Analyzing a clonal population of mouse CD4^+^CD25^+ ^T_Reg _cells, it was demonstrated that these cells do not proliferate in response to lymphopenia in the absence of the selecting self-peptide. This was in contrast to the naïve T cell proliferation behavior reflecting the lower IL-7 receptor (*IL7R*) expression levels in regulatory compared with naïve T cells [57], which was also supported by our data. Additionally, it was shown that GATA3 blocks *IL7R *expression in early stages of T cell development [58]. Because self-antigen presentation in combination with IL-7 expression promotes T_Reg _cell proliferation, we assume that this mechanism contributes to the specific accumulation of T_Reg _cells at sites where their self-antigen is presented.

Apart from the 'old friends', our T_Reg _cell signature comprises 41 'new players' that have not yet been described in T_Reg _cells at all. Because T_Reg _cells have a far-reaching effect on our health by influencing the outcome of infection, autoimmunity, transplantation, and cancer, we studied whether these new candidates have been reported to participate in these processes. Interestingly, the vast majority of the genes identified in our study (51 out of 62) have been implicated in at least one of these disease scenarios (Table 2).

### Genes involved in autoimmune diseases

Autoimmunity occurs as a consequence of self-tolerance breakdown, presumably resulting from a combination of inherited polymorphisms (or DNA variations), acquired environmental triggers, and stochastic events [59]. Analyzing our transcriptional pattern of human T_Reg _cells isolated from individual healthy donors, we found that 32 of the genes identified are involved in the pathogenesis of diverse autoimmune diseases (Table 2). We focus here on a few affected genes that are central to the functional modules discussed above and that might therefore influence disease pathogenesis.

We found *TNFRSF1B *to be 2.5-fold overexpressed in the naturally occurring T_Reg _cells compared with their naïve counterparts. A single nucleotide polymorphism (SNP) in this gene was reported to influence susceptibility to multiple sclerosis, a severe inflammatory autoimmune disorder of the central nervous system [60]. In addition, Sashio and coworkers [61] linked two other polymorphisms to the *TNFRSF1B *genelocus that increase susceptibility to Crohn's disease and ulcerative colitis, which are both chronic inflammatory diseases of the gastrointestinal tract. In Japanese patients, Morita and coworkers identified another SNP in the *TNFRSF1B *gene associated with systemic lupus erythematosus (SLE).

Type I diabetes is a T cell mediated inflammatory autoimmune disease of the endocrine pancreas, resulting in lack of insulin caused by β cell destruction. We found 18 genes in our human T_Reg _cell signature that have been reported to contribute to pathogenesis of this disease, including granzyme A (*GZMA*) [63], the CD40 ligand (*TNFSF5*) [64,65], *CTLA4 *[66], and the T-cell specific transcription factor 7 (*TCF7*) [67]. Furthermore, two polymorphisms in the *HLA-DRB1 *gene, which we found to be overexpressed in T_Reg _cells, have been described to confer high-risk susceptibility [68].

Rheumatoid arthritis (RA) is a chronic inflammatory disorder that affects the joints and is probably caused by autoimmune mechanisms. Twenty-one T_Reg _specific genes have been described as susceptibility genes for RA. For example, *LGALS3 *[69,70], *GZMA *[71], and the S100 calcium binding protein A4 (*S100A4*) [72] have been described as highly expressed in the synovial tissue and at sites of joint destruction contributing to the inflammatory process. The complex genetic component of RA etiology was further demonstrated by the discovery of multiple polymorphisms, for example in genes of the chemokine receptor 5 (*CCR5*) [73] and of *HLA-DRB1 *[74], conferring high risk susceptibility.

In mice deficient for *STAT4*, a gene we found to be downregulated in our human T_Reg _cells, RA is suppressed because of reduced levels of IL-12 and interferon interferon (IFN)-γ [75]. Interestingly, STAT4^-/- ^mice were additionally almost completely protected from diabetes [76] and induction of experimental allergic encephalomyelitis [77], underlining the importance of STAT4 downregulation in T_Reg _cells.

Because T_Reg _cells are essential for the maintenance of self-tolerance, SNPs or mutations that affect genes expressed in T_Reg _cells may result in the synthesis of aberrant mRNAs and proteins, which in turn could impair T_Reg _cell function and/or development, leading to higher risks for autoimmunity. Additionally, failures in gene regulation resulting in inadequate protein amounts could disturb appropriate T_Reg _cell activity, thereby probably contributing to the pathogenesis of autoimmune disorders.

Because most of the genes discussed here are central components of the functional modules discussed above, it is conceivable that the dysregulation of one or more of these genes affect T_Reg _cell activity in terms of survival/apoptosis, differentiation, proliferation, and suppressor function, thereby promoting breakdown of self-tolerance and eventually leading to autoimmunity.

## Conclusion

This study provides new insight into gene expression characterizing human regulatory versus naïve T cells from individual healthy donors. Based on our nonredundant microarray approach, we identified a comprehensive set of 62 'old friends' and 'new players' that are differentially expressed in T_Reg _cells. Pathway analysis implicated most of these genes in functional key modules of survival/apoptosis, TCR signaling/activation/proliferation, and differentiation/maintenance of T_Reg _cells and might therefore represent promising new targets for therapeutic intervention. This is underlined by the fact that these genes have been widely associated with diverse clinical setting of autoimmune diseases. Functional dissection of the modules under pathophysiological conditions should help to unravel the remaining mysteries of human T_Reg _cells and is essential for future development of new therapeutic approaches exploiting their potential in balancing peripheral tolerance.

## Materials and methods

### Blood samples from healthy donors

Blood samples were collected from 11 healthy donors after informed consent had been obtained, in accordance with institutional guidelines. The Ethics Committee of Hanover Medical School approved the study protocol. Basic characteristics of all donors are summarized in Table 1. None of the donors suffered from allergies or autoimmune disease and all were free from acute or chronic infections.

### Purification of human CD4^+ ^T cells

CD4^+ ^T cells were prepared from peripheral blood of healthy donors by centrifugation over Ficoll-Hypaque gradients (Biochrom AG, Berlin, Germany) and MACS isolation using the CD4^+ ^T cell isolation kit and AutoMACS technology (Miltenyi Biotech, Bergisch Gladbach, Germany). Subsequently, cells were separated into CD4^+^CD25^- ^and CD4^+^CD25^+ ^T cells by either using sorting on a MoFlo (DakoCytomation, Fort Collins, CO, USA) to a purity in excess of 98% (for Affymetrix studies) or an AutoMACS using the regulatory human T cell isolation kit (Miltenyi Biotech). To increase purity of the CD25^- ^T cell fraction an additional separation step depleting remaining CD25^+ ^T cells was added, if necessary. For studies on the Human T_Reg _Chip purity of the enriched cell fractions was above 90%, as determined by flow cytometry (the remaining contaminating cells mainly represent CD16^+^/CD56^+ ^natural killer cells and, at lower levels, CD8^+ ^T cells, CD19^+ ^B cells and CD14^+ ^monocytes; Additional data file 6). Isolated cells were either directly used for RNA purification or pooled equivalently as indicated before RNA purification.

### Purification of murine CD4^+ ^T cells

For Affymetrix GeneChip experiments, red blood cell depleted splenocytes from BALB/c mice were labeled with anti-CD4 and anti-CD25. Labeled cells were separated with a MoFlo and purity was in excess of 98%. Isolated cells were pooled equivalently (three independent individuals) and subsequently used for RNA purification.

### Propagation and stimulation of CD4^+ ^T cell lines

CD4^+^CD25^+ ^T_Reg _cells were stimulated once with plate-bound anti-CD3 (TR66, 1 μg/ml), soluble anti-CD28 (CD28.2, 1 μg/ml; BD Bioscience, San Jose, CA, USA), and 50 U/ml recombinant human IL-2 (Proleukin; provided by P Wagner, Chiron Corporation, Emeryville, CA, USA), and thereafter weekly with irradiated allogeneic EBV-transformed B cells (LG2-EBV; provided by T Boon, LICR, Brussels, Belgium). CD4^+^CD25^- ^T cells were stimulated directly with irradiated LG2-EBV cells. Culture medium was Iscove's modified Dulbecco's medium, with 10% fetal calf serum,100 U/ml penicillin/streptomycin, and nonessential amino acids (PAA Laboratories, Linz, Austria). Human peripheral blood was obtained after informed consent had been obtained, in accordance with institutional guidelines. Antibodies for immunostaining were PE-, FITC-, APC-, and CyChrom-conjugated antibodies against CD4 (RPA-T4), CD25 (M-A251; all from BD Bioscience), and FOXP3 (PCH101; eBioscine Inc., San Diego, CA, USA) and respective isotype controls. Anti-CD3ε (TR66, produced from hybridoma supernatants) and anti-CD28 (CD28.2; BD Bioscience) were used for T cell stimulation.

### Retroviral transduction of human effector CD4^+ ^T cells

The cDNA encoding human FOXP3 was amplified from cDNA of T_Reg _cells using high fidelity PFU polymerase (Promega) and specific primers (FOXP3: 5'-GAC AAG GAC CCG ATG CCC A-3' and 5'-TCA GGG GCC AGG TGT AGG GT-3'). The PCR product was cloned into pCR4.1 TOPO (Invitrogen, Carlsbad, CA, USA), sequenced, and inserted into a pMSCV-based retroviral vector encoding an enhanced GFP under the control of an IRES sequence. The amphotropic PT67 packaging cell line (provided by M. Wirth, GBF, Braunschweig, Germany) was used for transfection. Filtrated (0.45 μm) virus-containing supernatant supplemented with 8 mg/ml sequabrene (Sigma-Aldrich, Munich, Germany) was applied to T_h _cells at day 2 after allogeneic stimulation by centrifugation at 5000 × *g *for 60 minutes at room temperature. Cells were expanded thereafter with 50 U/ml IL-2, and GFP-expressing cells were sorted 1-2 weeks later using a FACS-Vantage (BD Bioscience).

### Flow cytometric analysis

To confirm purity of the separated cell fractions, regulatory and naïve T cells were analyzed by multicolor FACS using the following antibodies: anti-CD4-FITC and anti-CD25-PE (Miltenyi Biotec). Flow cytometry was done using a FACSCalibur applying CellQuest software (BD Bioscience).

### Real-time RT-PCR

CD4^+^CD25^+ ^regulatory and CD4^+^CD25^- ^naïve T cells were isolated by MACS technology as described above. After cell lysis, RNA was extracted from both cell populations applying the RNeasy kit (Qiagen, Hilden, Germany). cDNA was synthesized using oligo(dT) primers and random hexamers by SuperScript II Reverse Transcriptase (Invitrogen, Karlsruhe, Germany). Quantitative real-time RT-PCR was performed in an ABI PRISM cycler (Applied Biosystems, Foster City, CA, USA) using a SYBR Green PCR kit from Stratagene (La Jolla, CA, USA) and specific primers optimized to amplify 90-230 base pair fragments from the different genes analyzed. A threshold was set in the linear part of the amplification curve, and the number of cycles needed to reach it was calculated for every gene. Relative mRNA levels were determined by using included standard curves for each individual gene and further normalization to RPS9 as a housekeeping gene. Melting curves established the purity of the amplified band. Primer sequences are summarized in Table 3.

### Preparation of the Human T_Reg _Chip

A total of 395 oligonucleotides were deposited onto CodeLink activated slides (Amersham Biosciences, Freiburg, Germany) at a concentration of 25 μmol/l in 1.5× sodium phosphate buffer in a contact-dependent manner using a MicroGrid TAS II spotter (BioRobotics, Freiburg, Germany). All 50-mers were amino-modified at the 5'-end enabling covalent linkage to reactive ester groups provided by the glass surface. Coupling of DNA was ensured by overnight incubation in a saturated sodium chloride chamber, and blocking residual reactive groups was done as recommended by the manufacturer [78]. Until used, slides were maintained in a desiccated environment. To ensure complete spotting, SYBR-Green staining of three randomly selected Human T_Reg _Chips of each printing batch was performed as previously described [79].

### Design of the Human T_Reg _Chip

Each probe in our microarray consists of a single 50 mer oligonucleotide, because utility and performance of 50 mer oligonucleotide microarrays was previously established [80]. The Human T_Reg _Chip consists of 350 oligonucleotides probing genes specific for T_Reg _cells and 31 oligonucleotides representing housekeeping genes consulted for normalization. Furthermore, many control oligonucleotides are included: two 5'-3' controls to ensure RNA integrity, four bacterial hybridization controls to examine a linear hybridization process, five spike-in controls to check sample preparation, one positive control (*Arabidopsis thaliana*) for simpler grid finding, and finally 32 negative controls to calculate the background level. Altogether, we immobilize eight replicates per oligonucleotide, split into two separated arrays per slide, each containing 1,600 spots. Genes probed on the Human T_Reg _Chip were selected by extensive analyses of literature and previously conducted Affymetrix microarray experiments. Design and synthesis of the oligonucleotides were performed by MWG using the Affymetrix probe sets as reference. Our Human T_Reg _Chip will be made available to the scientific community on our website [81].

### Sample preparation, hybridization, washing, staining and scanning

Quality and integrity of the total RNA isolated from 1-2 × 10^5 ^CD4^+^CD25^+ ^and CD4^+^CD25^- ^T cells was controlled by running all samples on an Agilent Technologies 2100 Bioanalyzer (Agilent Technologies, Waldbronn, Germany). Samples were prepared by applying a double-linear amplification method in accordance with the Eberwine protocol[82] and modified by Affymetrix. Briefly, the first round of RNA amplification was performed without biotinylated nucleotides using the Promega P1300 RiboMax Kit for T7 amplification (Promega, Mannheim, Germany). After clean up of the precipitated aRNA synthesis of second round, first-strand cDNA was done using random hexamers (Pharmacia, Freiburg, Germany). Subsequent second-strand cDNA was prepared as in the first round but integrating an additional RNAse H incubation step to digest the aRNA before annealing of the T7T23V primer. The second round of RNA amplification was performed as an *in vitro *transcription assay in the presence of biotinylated UTP using the GeneChip^® ^Expression 3'-Amplification Reagents Kit for IVT Labeling (Affymetrix). The concentration of the obtained biotin-labeled cRNA was determined by ultraviolet absorbance and its quality as means of product length distribution was again checked using the Agilent Bioanalyzer. In all cases, 15 μg of each biotinylated cRNA preparation was fragmented and placed in a hybridization cocktail containing four biotinylated hybridization controls (BioB, BioC, BioD, and Cre). Samples were hybridized to individual Human T_Reg _Chips for 16 hours at 42°C using a Lucidea Slidepro (Amersham Biosciences). After hybridization the microarrays were washed as recommended in the manufacturer's instructions (CodeLink Expression Bioarray System; Amersham Biosciences), stained with Cy5-streptavidin (Amersham Biosciences), and read using an arrayWorX^e ^scanner (Applied Precision, Issaquah, WA, USA).

### Affymetrix GeneChip assay

Samples were amplified for GeneChip analysis according to the recommended protocols by the manufacturer. In all cases, 10 μg of each biotinylated cRNA preparation was fragmented and placed in a hybridization cocktail containing four biotinylated hybridization controls (BioB, BioC, BioD, and Cre), as recommended by the manufacturer. Samples were hybridized to an identical lot of Affymetrix GeneChips for 16 hours. After hybridization the GeneChips were washed, stained with SA-PE, and read using an Affymetrix GeneChip fluidic station and scanner.

### Criteria for * Human T_Reg _Chip *gene collection

Differentially expressed genes between CD4^+^CD25^+ ^and CD4^+^CD25^- ^measured on Affymetrix GeneChips were selected according to predefined categories deduced from three parameters calculated by MAS 5 software: fold change (FC), change *p v*alue (pValue), and signal intensity difference (SID). Category A is defined as an FC above 2, pValue <0.001 (for increased) or >0.999 (for decreased), and SID above 200. Category B is defined as FC above 2, pValue <0.01 (for increased) and >0.99 (for decreased), and SID above100. Category C is defined as FC above 1.5, pValue <0.001 (for increased) and >0.999 (for decreased), and SID above 40.

The likelihood of a significant regulation decreases from category A to C. Preferentially, most of the selected genes collected for the Human T_Reg _Chip are categorized as A. Selection was performed by collecting genes that were significantly regulated in human cells, genes that were similarly regulated between mouse and human, genes that were found to be regulated only in mouse cells and referenced in the literature, and genes that were significantly affected by FOXP3 overexpression in cultured T_h _cell lines. Also considered were genes known for their impact in mouse and human regulatory T cell development.

### Data analysis Human T_Reg _Chip

Signal intensities were qualified and quantified by means of Imagene software v5.5.2 (BioDiscovery, Los Angeles, CA, USA). Spots of poor quality (flag = 3) were excluded from further analysis. To adjust arrays from different experiments, data normalization based on median signal intensities of the housekeeping genes was carried out as proposed using the following formula:



Where SI_normalized _is the normalized signal intensity, I_n _is the mean signal intensity of gene n, B_n _is the mean background intensity of gene n, and <ln house> is the median signal intensity from housekeeping genes expressed as ln (logarithm naturalis).

Differences in gene expression among CD4^+^CD25^+ ^regulatory and CD4^+^CD25^- ^naïve T cells were determined statistically by corrected *t *test analysis using the SAM tool [83]. Differentially expressed genes were defined using the following SAM parameters: delta = 2.46 and median FDR (false discovery rate) = 0.48. For two-dimensional hierarchic clustering analysis Genesis software v1.4.0 was applied [84].

### Accession numbers

The entire data sets are deposited in a MIAME compliant format at Gene Expression Omnibus (GEO) [85]. Data derived from the Human T_Reg _Chip are available under the series accession number GSE3882 (platform ID, GPL3110).

Data derived from Affymetrix GeneChip system and used as reference and selection data sets are published at GEO under series accession number GSE4527 (FOXP3 and GFP transduced CD4^+ ^T_h _cells) and GSE4571 (representing data from CD4^+^CD25^+ ^and CD4^+^CD25^- ^T cellsisolated by cell sorting from human peripheral blood and CD4^+^CD25^+ ^and CD4^+^CD25^- ^T cells isolated by cell sorting from spleen prepared from BALB/C mice).

## Additional data files

The following additional data are available with the online version of this paper: An Excel spreadsheet containing lists of differentially expressed genes in murine and human CD4^+^CD25^+ ^T cells versus CD4^+^CD25^- ^T cells obtained from whole-genome Affymetrix GeneChips (Additional file 1); an Excel spreadsheet containing a list of genes that were likewise affected by Foxp3 overexpression in CD4^+ ^T_h _cell lines and CD4^+^CD25^+ ^derived T_Reg _cell lines compared with their appropriate controls (data obtained using whole-genome Affymetrix GeneChip HG-U133A; Additional data file 2); an Excel spreadsheet containing a list of known genes that were previously discussed in the literature within the context of human and murine regulatory T cells (Additional data file 3); an Excel spreadsheet containing relative expression datafrom Foxp3 overexpressing CD4^+ ^T_h _cell lines versus their GFP tranduced CD4^+ ^T_h _controls obtained from whole genome Affymetrix GeneChip HG-U133A (data are presented for genes that are also accessible on the Human T_Reg _Chip; Additional data file 4); a Word file presenting data for the regulatory phenotype and the amount of Foxp3^+ ^cells within MACS purified human CD4^+^CD25^+ ^T cells (Additional data file 5); and a Word table describing the phenotype of contaminating cells within MACS purified CD4^+^CD25^+ ^and CD4^+^CD25^- ^T cells (Additional data file 6).

## Supplementary Material

Additional File 1 Click here for file

Additional File 2 Click here for file

Additional File 3 Click here for file

Additional File 4 Click here for file

Additional File 5 Click here for file

Additional File 6 Click here for file

## Figures and Tables

**Table 1:** Characteristics of healthy volunteers

Donor	Age	Sex
A	58	Male
B	57	Female
C	27	Female
D	27	Female
E	36	Male
F	39	Male
G	39	Male
H	26	Female
I	62	Female
J	54	Female
K	26	Male

**Table 2:** Genes differentially expressed in human CD4^+^CD25^+ ^regulatory *vs *CD4^+^CD25^- ^naive T cells

Gene symbol	Gene name	Autoimmunity
*NINJ2*	Ninjurin 2	
*ACTN1*	Actinin, alpha 1	SLE, CHA
*NELL2*	NEL-like 2	
*ITGB2*	Integrin, β_2_	UC, MC, COPD, T2D, AS, LAD-1, RA, ALPS, SLE
*TIAF1*	TGFB1-induced antiapoptotic factor 1	
*TP53INP1*	Tumor protein p53 inducible nuclear protein 1	
*TRAF1*^a^	TNF receptor-associated factor 1	
*LGALS1*	Galectin 1	JIA, RA, IBD
*LGALS3*^a^	Galectin 3	RA, JIA
*GZMA*	Granzyme A	T1D, RA, SLE, IBD
*GZMK*	Granzyme K	
*PTTG1*	Pituitary tumor-transforming 1	Diabetes
*TRIB1*	Tribbles homolog 1	
*S100A10*	S100 calcium binding protein A10	
*CEB1*	Hect domain and RLD 5	
*SLAMF1*	Signaling lymphocytic activation molecule family member 1	SLE, X-linked XLP, RA, MS
*S100A4*	S100 calcium binding protein A4	RA
*PIM1*	Pim-1 oncogene	
*ID2*	Inhibitor of DNA binding 2	Diabetes
*FHIT*	Fragile hisT1Dine triad gene	
*RBMS1*	RNA binding motif, single stranded interacting protein 1	
*IFITM1*	Interferon induced transmembrane protein 1	
*IL2RA*^a^	Interleukin 2 receptor, alpha	T1D, profound cellular immunodefiency
*TNFRSF1B*^a^	Tumor necrosis factor receptor superfamily, member 1B	MC, UC, MS, SLE
*CCR5*	Chemokine (C-C motif) receptor 5	MS, Grave's disease, RA
*GPR2*	Chemokine (C-C motif) receptor 10	Autoimmune skin diseases
*IL2RB*	Interleukin 2 receptor, beta	ITP, RA, osteoarthritis, hemolytic anemia
*G1P2*	Interferon, alpha-inducible protein	
*IL1RL2*	Interleukin 1 receptor-like 2	
*IL7R*	Interleukin 7 receptor	SCID, RA, SLE
*CCR7*^a^	Chemokine (C-C motif) receptor 7	Diabetes, SLE, MS, RA, JIA
*TNFSF5*	CD40 ligand (TNF superfamily, member 5, hyper-IgM syndrome)	HIGM1, Alzheimer disease, T1D, SLE, MS, AS, ITP
*CCL5*	Chemokine (C-C motif) ligand 5	EAT, MS, diabetes, SLE, RA
*TNFRSF10B*	Tumor necrosis factor receptor superfamily, member 10b	MS, RA
*SDC4*	Syndecan 4	
*CTLA4*^a^	Cytotoxic T-lymphocyte-associated protein 4	T1D, Grave's disease, SLE
*TFRC*	Transferrin receptor	
*AKAP2*	A kinase (PRKA) anchor protein 2	
*DGKA*	Diacylglycerol kinase, alpha	
*PITPNC1*	PhosphaT1Dylinositol transfer protein, cytoplasmic 1	
*TRGV9*	T cell receptor gamma variable 9	
*CD81*	CD81 antigen	
*PECAM1*	Platelet/endothelial cell adhesion molecule	ITP, diabetes, AS, RA, CIA, MS
*FOXP3*	Forkhead box P3	IPEX, T1D
*GATA3*	GATA binding protein 3	RA, HDR syndrome
*BHLHB2*	Basic helix-loop-helix domain containing, class B, 2	SLE
*SATB1*	Special AT-rich sequence binding protein 1	
*STAT4*	Signal transducer and activator of transcription 4	MC, EAE, UC, diabetes, COPD, SLE, arthritis
*STAT6*	Signal transducer and activator of transcription 6	EAE, RA, autoimmune uveitis, diabetes
*MYC*	v-myc Myelocytomatosis viral oncogene homolog	Diabetes, RA, SLE
*TCF7*	Transcription factor 7 (T-cell specific, HMG-box)	T1D
*XBP1*	X-box binding protein 1	T2D, RA
*CNOT2*	CCR4-NOT transcription complex, subunit 2	
*HLA-DMA*	Major histocompatibility complex, class II, DM alpha	T1D, SLE, RA
*HLA-DRB1*	Major histocompatibility complex, class II, DR beta 1	RA, MS, sarcoidosis, Sjögren's syndrome, Grave's disease, T1D
*HLA-DRB3*	Major histocompatibility complex, class II, DR beta 3	SLE, RA, MS, sarcoidosis, Sjögren's syndrome, Grave's disease
*GBP2*	Guanylate binding protein 2, interferon-inducible	
*GBP5*	Guanylate binding protein 5	
*SLC40A1 *(*a*)	Solute carrier family 40 (iron-regulated transporter), member 1	
*SHMT2 *(*b*)	Serine hydroxymethyltransferase 2 (mitochondrial)	
*EPSTI1*	Epithelial stromal interaction 1	
*NOSIP*	Nitric oxide synthase interacting protein	

^a^Genes that were additionally found to be induced upon retroviral over-expression of FOXP3 in CD4^+^CD25^- ^T cells. ALPS, autoimmune lymphoproliferative syndrome; AS, atherosclerosis; CHA, autoimmune chronic active hepatitis; CIA, collagen-induced arthritis; COPD, chronic obstructive pulmonary disease; EAE, experimental autoimmune encephalomyelitis; EAT, experimental autoimmune thyroiditis; HIGM1, hyper-IgM immunodefiency syndrome type I; IPEX, immunodysregulation, polyendocrinopathy, and entheropathy, X-linked; JIA, juvenile idiopathic arthritis; IBD, inflammatory bowel disease; ITP, idiopathic thrombocytopenic purpura; LAD-1, leukocyte adhesion deficiency-1; MC, Morbus Crohn; MS, multiple sclerosis; RA, rheumatoid arthritis; SCID, severe combined immunodefiency; SLE, systemic lupus erythematosus; T1D, type I diabetes; T2D, type II diabetes; UC, ulcerative colitis; XLP, X-linked lymphoproliferative syndrome.

**Table 3:** Primer sequences used in real-time RT-PCR

Gene	Primers
*FOXP3*	5'-GAA CGC CAT CCG CCA CAA CCT GA-3'
	5'-CCC TGC CCC CAC CAC CTC TGC-3'
*CTLA4*	5'-TGC AGC AGT TAG TTC GGG GTT GTT-3'
	5'-CTG GCT CTG TTG GGG GCA TTT TC-3'
*CCR7*	5'-TGG CCT GCA GGA AAC ACC-3'
	5'-GGG AGA CTT CTT GGC TTG GTG AG-3'
*RPS9*	5'-CGC AGG CGC AGA CGG TGG AAG C-3'
	5'-CGA AGG GTC TCC GCG GGG TCA CAT-3'
*TNFRSF1B*	5'-GTA GCC TTG CCC GGA TTC TGG-3'
	5'-ACC CTG CCC CTG CTC TGC TA-3'
*TRAF1*	5'-GGG GCA TAA ACT TTC CTC TTC C-3'
	5'-TTT GGG GTT ATA CAT TGC TCA GTG-3'
*LGALS3*	5'-CCT TTG CCT GGG GGA GTG GTG-3'
	5'-TGA AGC GTG GGT TAA AGT GGA AGG-3'

RT-PCR, reverse transcription polymerase chain reaction.
