# Supplementary material for: Signatures of human regulatory T cells: an encounter with old friends and new players
Source: Genome Biol. 2006 Jul 12;7(7):R54. doi: 10.1186/gb-2006-7-7-r54 (PMC1779567; doi:10.1186/gb-2006-7-7-r54)
Supplement: Additional File 5 [file gb-2006-7-7-r54-S5.doc]

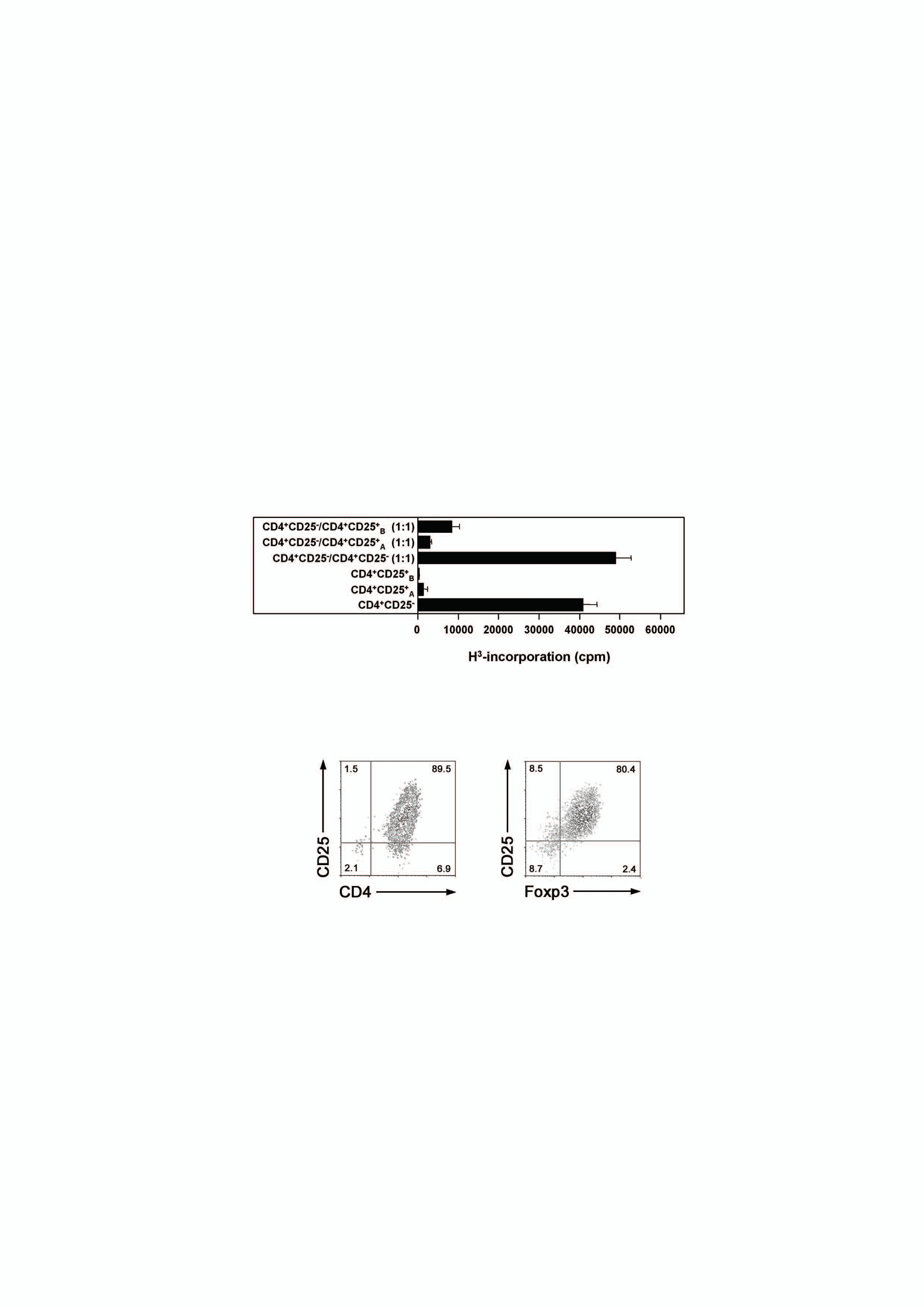


**Supplement Figure1. Regulatory phenotype of MACS purified human CD4+CD25+ T cells**

Isolation of CD4^+^CD25^+^ T cells by MACS enriches functional and phenotypic CD4^+^CD25^+^ T_Reg_ cells. (Upper panel) CD4^+^CD25^-^ and CD4^+^CD25^+^ derived T cells were stimulated with allogeneic EBV B cells alone or in the presence of equal numbers of indicated T cells. Proliferation was assessed at day 3, background proliferation was 891.3 +/-376.2 cpm. (Lower panel) CD4^+^CD25^+^ derived T cells were analyzed for cell surface CD4 (RPA-T4, BD Bioscience) and CD25 (clone M-A251, BD Bioscience) expression and intra-nuclear FOXP3 expression (clone PC101, eBioscience). Percent of positive cells is indicated. Data shown is representative for three independent experiments.
