# Supplementary material for: Signatures of human regulatory T cells: an encounter with old friends and new players
Source: Genome Biol. 2006 Jul 12;7(7):R54. doi: 10.1186/gb-2006-7-7-r54 (PMC1779567; doi:10.1186/gb-2006-7-7-r54)
Supplement: Additional File 6 [file gb-2006-7-7-r54-S6.doc]

|  | **phenotype of contaminating cells within** | |
| --- | --- | --- |
|  | **CD4^+^CD25^+^** | **CD4^+^CD25^-^** |
| **CD14** | 10.4 / 11.4 / 9.8 | 3.9 / 5.6 / 8.9 |
| **CD19** | 8.2 / 8.0 / 3.4 | 3.2 / 3.2 / 4.5 |
| **CD56/CD16** | 77.3 / 73.6 / 77.7 | 86.8 / 89.0 / 83.9 |
| **CD8** | 4.1 / 7.0 / 10.0 | 6.1 / 2.2 / 2.7 |

**Supplement Table 5:** Phenotype of contaminating cells within MACS purified CD4^+^CD25^+^ and CD4^+^CD25^-^ T cells. Data was obtained from three healthy donors and re-analysed by FACS. The absolute number of contaminating cells was set to 100% and percentages of surface marker expression are shown.
